# Mothers with Concurrent Opioid and Cocaine Use and Neonatal Opioid Withdrawal Syndrome

**DOI:** 10.3390/children12070916

**Published:** 2025-07-11

**Authors:** Divya Rana, Linda DeBaer, Massroor Pourcyrous

**Affiliations:** 1 Department of Pediatrics, The University of Tennessee Health Science Center, Memphis, TN 38103, USA; ldebaer@uthsc.edu (L.D.); mpourcyr@uthsc.edu (M.P.); 2 Department of Obstetrics & Gynecology, The University of Tennessee Health Science Center, Memphis, TN 38103, USA; 3 Department of Physiology, The University of Tennessee Health Science Center, Memphis, TN 38103, USA

**Keywords:** maternal opioids with and without cocaine use, polysubstance, fentanyl use in pregnancy with neonatal outcomes and neonatal opioid withdrawal syndrome

## Abstract

Background: Polysubstance use, particularly combining opioids with stimulants such as cocaine, is rising among individuals with substance use disorders. This practice aims to balance cocaine’s stimulant effects with opioids’ sedative effect, potentially decreasing adverse outcomes. We hypothesized that concurrent exposure to cocaine and opioids would reduce the risk of neonatal opioid withdrawal syndrome (NOWS) compared to opioid use alone. Methods: This analysis draws from an ongoing prospective study of maternal substance use (SUD) at Regional One Health’s perinatal center in Memphis, TN, and included mothers and their infants born between 2018 and 2022. Maternal SUD was identified via screening questionnaires, urine toxicology, or umbilical cord tissue analysis. Participants were grouped into using (a) opioids with cocaine (OwC) and (b) opioids without cocaine (OwoC). Univariate and regression analyses were conducted to assess the risk of NOWS. Results: A total of 353 infants were born to 342 mothers, with 31% (110/353) of the infants born to women who used cocaine along with opioids. While maternal demographics were similar, the OwC group had significantly lower rates of prenatal care, chronic pain history, and MOUD enrollment (*p* = 0.03). Infants in the OwC group had significantly higher rates of NOWS (*p* < 0.01), longer hospital stays (*p* < 0.01), and 6.5 times greater odds of developing NOWS (*p* < 0.001). NOWS was associated with an average 15-day increase in the length of stay for term infants (95% CI: 11.2, 18.8; *p* < 0.001). Conclusions: Contrary to our hypothesis, our study highlights the significant impact of maternal cocaine use on the increased likelihood of NOWS and extended hospital stays for affected infants.

## 1. Introduction

Substance use during pregnancy remains a critical public health issue, with evolving patterns of multiple or polysubstance maternal drug exposure [1,2]. Historically, opioid use disorder (OUD) has been a predominant concern due to its association with neonatal opioid withdrawal syndrome (NOWS). In the last decade, there has been a rise in the use of illicit synthetic opioids, like fentanyl, as prescription opioid use declined [3]. During this time, there has been a growing trend of polysubstance use. Between 2013 and 2016, the rate of cocaine-related overdoses in combination with fentanyl among U.S. adults doubled [4,5,6,7]. Similar trends have been reported during pregnancy, particularly the concurrent use of opioids and stimulants such as cocaine or methamphetamine [8,9]. This pattern is especially concerning in the era of illicit fentanyl, which has largely displaced prescription opioids and increased the risks associated with prenatal substance exposure.

The simultaneous use of fentanyl and stimulants is driven by complex behavioral and neurochemical interactions. Cocaine, a powerful stimulant, enhances alertness, euphoria, and energy levels by increasing dopamine availability in the brain [10,11]. In contrast, fentanyl, a synthetic opioid, induces sedation, respiratory depression, and analgesia. Some individuals co-use these substances to balance their psychoactive effects, potentially modulating the intensity of opioid-induced sedation while prolonging the euphoric state [12]. The combination of opioids and cocaine, known as “speedballs,” produces a unique profile of subjective effects that may contribute to its appeal among users, and there also may be the perception that co-use potentially reduces the risk of respiratory suppression [13]. However, the pharmacologic complexity of fentanyl, combined with polysubstance use, remains largely unknown at present. This practice does increase the risk of unpredictable drug interactions, overdose, and adverse perinatal outcomes [14]. In pregnant women, stimulant–opioid co-use may exacerbate maternal cardiovascular stress, placental dysfunction, and fetal hypoxia, further complicating neonatal health outcomes [9,15]. To date, relatively little has been published on concomitant substance exposure during pregnancy, as most studies have focused primarily on opioid use alone.

Our study aims to strengthen the current evidence base by examining the impact of these combined exposures. This study is grounded in a translational framework that connects maternal substance use patterns—particularly opioid and stimulant co-use—to neonatal outcomes, with a focus on the risk of neonatal opioid withdrawal syndrome. The implications extend beyond assessing short-term risks such as NOWS, feeding difficulties, and neuroimaging findings, to understanding potential long-term neurodevelopmental outcomes in these children.

In this study, we sought to evaluate the relationship between maternal cocaine use and the incidence and severity of NOWS in neonates exposed to opioids. We hypothesized that cocaine co-exposure would reduce the risk of NOWS due to its stimulant properties, counteracting opioid effects.

## 2. Materials, Subjects, and Methods

This is a retrospective observational study conducted at the main campus of Regional One Health (ROH). The current data utilized de-identified ancillary data collected from a prospective, ongoing institutional study on maternal SUDs (MSUDs) and their infants, conducted at our tertiary perinatal center’s level III neonatal intensive care unit (NICU). ROH is a county-owned acute care hospital located in the medical district of Memphis, Tennessee, with a tertiary perinatal center and level III neonatal intensive care unit (NICU) that serves high-risk pregnancies and low-income mothers. This study was dually approved by the Institutional Review Board (IRB) of the University of Tennessee Health Science Center (UTHSC) and the ROH Office of Medical Research. IRB approval was also obtained to waive informed consent.

### 2.1. Study Population

This study included neonates born between 2018 and 2022 to mothers with confirmed opioid use determined by self-reporting, urine screening, or umbilical cord tissue toxicology (UCT).

Inclusion criteria: Mothers and infants with active or recent maternal opioid use during pregnancy. “Substance use” encompassed the use of amphetamine, cocaine, heroin, tobacco, marijuana, alcohol, other opioids, and benzodiazepines. Exclusion criteria included women who received opioids, benzodiazepines, or fentanyl by any route (oral, intravenous, intramuscular, epidural, or other) for pain management during labor and delivery, including use with anesthesia and prior to placental separation.

Since 2007, the ROH NICU has established center-specific consensus guidelines for managing newborns exposed to opioids in utero. All newborns with prenatal opioid exposure are admitted to the NICU (open unit includes special care, intermediate care, and intensive care) for observation of NOWS for 5–7 days or longer if necessary [14]. Assessment of infants at risk of NOWS was performed by using the modified Finnegan scoring (mFS) method. Non-pharmacological methods like a quiet ambient environment, swaddling, cohorting, massages, etc., are universally utilized for all opioid-exposed infants prior to and during pharmacological intervention. Parents are encouraged to be actively involved in the care of their infants to assess neonatal withdrawal and be active participants in treatment strategies [9,10]. These guidelines remained unchanged during the study period. Although our institution supports the use of the Eat, Sleep, and Console (ESC) approach for managing NOWS in term infants, the majority of opioid-exposed infants in our cohort were late preterm or early term. Because ESC has not been validated for use in these populations—who often demonstrate developmental immaturity and atypical withdrawal presentations—the modified Finnegan Score (mFS) was used consistently for assessment and treatment decisions throughout the study period.

We defined “severe NOWS” as requiring pharmacological treatment, whereas “mild or no NOWS” referred to cases not requiring such interventions, using a standardized definition which incorporates elements of mFS [16,17]. For the purposes of data presentation, NOWS was operationalized as the need for pharmacological treatment. Treatment for severe NOWS was initiated based on a modified Finnegan score (mFS) exceeding 8 in three consecutive scoring intervals or exceeding 12 in two consecutive intervals. Oral morphine sulfate was used as the first line of treatment. A morphine dose was given with each feeding every 4 h with a starting dose of 0.02 mg/kg/dose and a maximum dose of 0.2 mg/kg/dose. The dose tapering was initiated within 24 to 48 h of stable mFS. When the morphine dose reached 0.12 mg/kg/dose, secondary treatment was started with oral phenobarbital 2.5 mg/kg/dose every 12 h. Secondary treatment could be initiated prior to reaching the maximum morphine dose depending on the clinical presentation.

### 2.2. Outcomes and Variables

The primary outcome variable for the study was the risk of severe NOWS requiring pharmacological treatment. Cocaine exposure was the indicator variable. The study population differences were based on maternal self-reports of cocaine/opioid use, stratified by their concomitant use. Variables collected included data on medications utilized for opioid use disorder (MOUD) therapy, including methadone or buprenorphine; the treatment duration at delivery; maternal and infant demographic data; NOWS diagnosis and treatment; type of feedings; discharge weight; and length of stay, which was defined as the time to home discharge (rather than readiness of home discharge) to account for clearance from the State of Tennessee Department of Child Services (DCS). Race and ethnicity were recorded as reported by the mother and included Hispanic or non-Hispanic, Black or African American, White or Caucasian, and Asian or Native American as other.

### 2.3. Statistical Analysis

The primary comparison was based on maternal reporting of opioids with and without cocaine. Participants were categorized into two groups for comparative analysis, namely those who used (a) opioids with cocaine (OwC) and (b) opioids without cocaine (OwoC). Descriptive statistics were used to summarize demographic and clinical variables, stratified by substance use groups. Continuous variables were reported as mean ± standard deviation (SD) or median with interquartile range (IQR), depending on data distribution, and categorical variables were summarized as counts and percentages.

Group differences for categorical variables were assessed using robust chi-squared tests to account for potential violations of expected cell counts. For continuous variables, two-sample t-tests were used for normally distributed data, and the Mann–Whitney U test was applied for non-normally distributed variables.

Multivariable logistic regression was performed to examine the association between neonatal opioid withdrawal syndrome (NOWS) and maternal substance use, adjusting for key confounders including gestational age at birth, preterm birth (PTB), polysubstance use, and prenatal care (PNC) status. Odds ratios (ORs) and 95% confidence intervals (CIs) were reported. Predicted probabilities of NOWS were calculated to explore the effects of opioid use categories while holding other covariates constant.

To evaluate the factors associated with neonatal length of stay (LOS), a multivariable linear regression model was constructed. Independent variables included gestational age, NOWS diagnosis, maternal cocaine–opioid co-use, and the interaction between preterm birth and NOWS. Coefficients were interpreted as the estimated change in LOS (in days) for each unit or category of the predictor variable. Model fit was assessed using R^2^ and root mean square error (RMSE). An alpha level of 0.05 was used to determine statistical significance. All analyses were conducted using STATA^®^ version 18.0 (StataCorp, College Station, TX, USA).

## 3. Results

Among the 342 mothers in the MSA study, 32% (108/342) with 31% (110/353) of their newborns were exposed to both opioids and cocaine (OwC), while the remaining 69% (234/342) used opioids without cocaine (OwoC). The two groups were comparable in maternal age, race, education level, and insurance status. However, significantly fewer women in the OwC group reported adequate prenatal care (64% vs. 82%, *p* < 0.01). Additionally, women in the OwC group were less likely to be enrolled in medication for opioid use disorder (MOUD) (26% vs. 39%, *p* = 0.03) and had lower rates of chronic pain diagnoses (5% vs. 29%, *p* < 0.01) (Table 1).

Polysubstance use was prevalent in both groups, but women in the OwC group had significantly higher rates of illicit fentanyl (*p* < 0.01), marijuana (*p* < 0.01), and overall, a greater number of them engaged in substance use (*p* < 0.01). On average, women in the OwC group had five substances compared to three in the opioid-only group (*p* < 0.01). There were no significant differences in the rates of amphetamine, heroin, or benzodiazepine use, as well as the maternal psychiatric disorder history between the groups.

Neonatal characteristics were largely comparable between the groups (Table 2), with similar mean gestational ages at birth (36 ± 4 vs. 36 ± 3 weeks, *p* = 0.42) and head circumferences (31 ± 3 cm vs. 32 ± 3 cm, *p* = 0.16). However, there was a trend toward lower birth weights among infants exposed to opioids with cocaine (2512 ± 712 g vs. 2656 ± 763 g, *p* = 0.09). There was a trend toward higher preterm birth rates in the OwC group (47% vs. 37%, *p* = 0.06). Infants in the OwC group had significantly worse postnatal outcomes. They were more likely to require pharmacologic treatment for neonatal opioid withdrawal syndrome (NOWS) (56% vs. 35%, *p* < 0.01) and had prolonged hospital stays (28 ± 22 days vs. 21 ± 21 days, *p* < 0.01). Infants exposed to both cocaine and opioids (OwC group) had significantly lower discharge weights compared to those in the opioid-only group (OWoC) (2619 ± 773 g vs. 3060 ± 810 g, *p* < 0.01). Despite these differences, rates of abnormal head ultrasound findings were similar between groups (31% vs. 32%; *p* = 1.00). In addition, OwC-exposed infants were less likely to be discharged home with their mothers (13% vs. 65%, *p* < 0.01).

**Figure 1:**
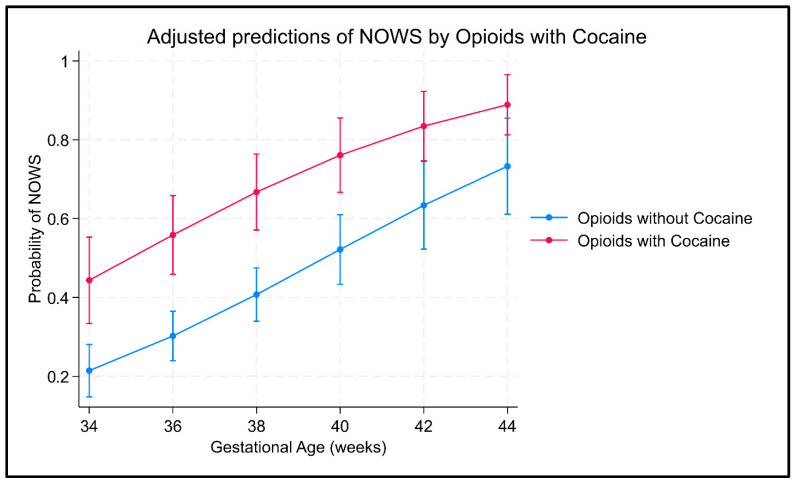
Risk of NOWS with and without prenatal cocaine exposure.

Multivariable logistic regression analysis revealed that infants in the OwC group had significantly increased odds of NOWS diagnosis by nearly 6.5 times (adjusted OR: 6.51; 95% CI: 2.73–15.49; *p* < 0.001), even after adjusting for potential confounders such as gestational age, polysubstance use, illicit fentanyl use, and MOUD enrollment. We calculated the predicted probabilities of NOWS at various gestational ages at birth (≥34 weeks), comparing the groups, which illustrated the increased risk with use of opioids with cocaine (OWC) than opioids alone (OWoC) (Figure 1).

Length of hospital stay (LOS) varied by gestational age and substance exposure (Supplemental Table S1). Among term infants, those exposed to both opioids and cocaine had a significantly longer LOS compared to those exposed to opioids alone (*p* = 0.02). In preterm infants treated for NOWS, LOS was also significantly longer in the opioid and cocaine group (*p* = 0.02), while other comparisons did not reach statistical significance. In the multivariable linear regression model, several factors were significantly associated with length of hospital stay (LOS). A diagnosis of NOWS was strongly associated with longer hospital stays (infants diagnosed with NOWS stayed about 26 days longer in the hospital; 95% CI: 21.04 to 30.16, *p* < 0.001) across all gestational ages. After adjusting for PTB (<37 weeks), NOWS remained a significant predictor, associated with an average increase of approximately 15 days in LOS (95% CI: 11.2, 18.8; *p* < 0.001).

## 4. Discussion

Contrary to our initial hypothesis, maternal use of opioids with cocaine (OwC) was associated with a 6.5-fold increased risk of neonatal opioid withdrawal syndrome (NOWS), independent of other factors such as gestational age, polysubstance use, and enrollment in medication for opioid use disorder (MOUD). This challenges the assumption that the stimulant properties of cocaine might counteract the sedative effects of opioids and mitigate the severity of NOWS. However, polysubstance use, including illicit fentanyl, was also more prevalent among mothers who reported concurrent opioid and cocaine use. Fentanyl, a highly potent synthetic opioid approximately 100 times stronger than morphine, poses a significantly higher risk for severe neonatal opioid withdrawal syndrome (NOWS).

Additionally, maternal concurrent opioid and cocaine (OwC) use were linked to significantly longer hospital stays, lower discharge weights, and a decreased likelihood of infants being discharged home with their mothers. These findings show the intricate dynamics of polysubstance use during pregnancy—particularly in the context of widespread illicit fentanyl exposure—increasing fetal and neonatal risks, highlighting the continued need for integrated maternal engagement in substance use treatment programs early in pregnancy along with enhanced support services to improve prenatal care and neonatal outcomes.

Our findings align with previous studies on maternal characteristics following prenatal exposure to cocaine [18] and opioids [19]. We saw less engagement in prenatal care, MOUD, and higher polysubstance use in the OwC cohort. The Maternal Lifestyle Study (MLS) [20] provides valuable insights into the consequences of prenatal cocaine and opioid exposure. This study found that mothers who were exposed to cocaine and opioids were at a significantly higher risk for various medical complications, including syphilis, gonorrhea, hepatitis, and placental abruption. The odds of psychiatric, nervous, and emotional disorders were also elevated, as was the likelihood of experiencing violence during pregnancy. Furthermore, studies have also found that women with substance use disorders are less likely to receive adequate prenatal care, increasing the risk of preterm birth, low birth weight, and other adverse neonatal outcomes [18,21].

We observed higher rates of preterm birth and low birth weight among infants born to mothers in the OwC group, though these differences did not reach statistical significance. Independently, both cocaine and opioid use during pregnancy are associated with serious maternal and neonatal complications. Prenatal cocaine exposure has been linked to adverse outcomes such as preterm birth, low birth weight, and congenital cardiac anomalies [22]. Additional perinatal risks reported in the literature include the premature rupture of membranes, reduced head circumference [23], and fetal demise [24], further supporting the trends observed in our cohort [25,26,27]. Chronic prenatal exposure to cocaine has been shown to alter cerebrovascular responses in newborn animal models, suggesting potential long-term impacts on neurovascular regulation [28,29]. No significant differences were observed in birth head circumference or head ultrasound findings between the exposure groups. However, explicit neurological outcomes were not assessed in this study.

Similarly, intra-partum opioid use, including prescription opioids, may also result in preterm birth and a low birth weight in addition to NOWS, a condition requiring prolonged hospitalization and pharmacologic treatment [17,30]. In recent years, the landscape of opioid use during pregnancy has shifted, with a decline in prescription opioid use and a concurrent rise in synthetic opioids. The increasing prevalence of illicit fentanyl—often used in combination with stimulants such as cocaine and methamphetamine—has heightened the risk of polysubstance use and in utero fetal exposure, making this a more pressing concern in contemporary perinatal care. This co-use pattern is thought to balance the opposing psychoactive effects of these substances, with cocaine’s stimulant properties counteracting opioid-induced sedation [31]. However, this practice may have unintended consequences, with increase in overdose death [32].

In our study, we report a higher risk for NOWS among infants with concurrent opioid and cocaine exposure. Our findings align with national data showing that polysubstance exposure, particularly combined exposure to cocaine and heroin, has been reported to exacerbate neonatal withdrawal severity, leading to increased rates of pharmacologic intervention and longer hospital stays [20,33]. Prior research has also linked such exposures with fetal growth restriction and neurodevelopmental deficits that can persist in childhood [34,35]. The heightened severity of withdrawal may be attributed to the distinct pharmacologic actions of these substances; for instance, cocaine’s stimulant effects may initially mask opioid withdrawal symptoms, followed by a delayed and intensified withdrawal phase [36].

Despite these insights, contemporary data on the combined effects of synthetic high-potency opioids, such as fentanyl and cocaine, remain limited. Our previous work has demonstrated increased NOWS severity associated with prenatal fentanyl exposure, underscoring the urgent need for updated clinical guidelines that reflect evolving patterns of substance use in pregnancy [37,38]. Several mechanisms may underlie the increased risk observed. Cocaine has been shown to impair placental function, potentially enhancing fetal opioid exposure by disrupting placental drug metabolism [39,40]. Additionally, the higher prevalence of illicit fentanyl use in the cocaine-exposed group (*p* < 0.01) may contribute to the observed increase in NOWS severity, given fentanyl’s association with more prolonged and severe withdrawal courses.

A notable issue is the potential confounding effect of prenatal cocaine exposure on neonatal neurobehavior. Symptoms such as jitteriness or hypertonicity may mimic signs of opioid withdrawal, complicating clinical assessment [36]. In practice, distinguishing between these overlapping presentations can be challenging. As such, treatment for NOWS is often initiated based on the infant’s clinical trajectory to ensure timely and appropriate care [41].

Furthermore, the prolonged hospitalization seen in this group may be attributed to more severe withdrawal symptoms requiring extended pharmacologic treatment as well as a potential delay related to home disposition and safety measures needed in such circumstances. This increase in length of stay (LOS) remained significant even after adjusting for gestational age, suggesting that prematurity alone does not account for the observed differences. Additionally, infants in this group were more likely to require longer treatment durations and adjunctive therapy with phenobarbital, indicating a more complex and refractory withdrawal course. These findings are consistent with prior studies demonstrating that polysubstance exposure, particularly involving stimulants like cocaine and illicit fentanyl, is associated with an increased severity of neonatal opioid withdrawal [17,30,38]. Additionally, the lower rates of maternal prenatal care (*p* < 0.01) and MOUD enrollment (*p* = 0.03) in the OwC group suggest that these infants may have experienced more in utero instability, further contributing to poorer outcomes.

Clinical Implications and Future Directions: These findings underscore the urgent need to address the growing issue of polysubstance use during pregnancy, particularly the co-use of opioids with cocaine and other stimulants. Standard protocols for managing neonatal opioid withdrawal syndrome (NOWS) may require adaptation for infants exposed to both opioids and stimulants, as their withdrawal symptoms and treatment responses may differ from those exposed to opioids alone. Further research is essential to better understand these differences and guide evidence-based care. Additionally, improving access to prenatal care and MOUD for individuals using cocaine could help mitigate some of the adverse neonatal outcomes observed in this study. Future research should focus on elucidating the precise mechanisms by which cocaine exacerbates NOWS and identifying optimal treatment approaches for this high-risk population. Given the increasing prevalence of polysubstance use in pregnancy, ongoing surveillance and policy efforts are needed to improve maternal and neonatal health outcomes in this vulnerable population. We observed that infants in the opioid-with-cocaine (OWC) group had lower birth weight and lower discharge weights. However, the proportion of small-for-gestational-age (SGA) status was not assessed among groups. Although we were not able to examine the underlying factors contributing to this discrepancy within the scope of this study, this finding highlights an important area for future research.

Study Limitations: This study has several limitations that should be considered when interpreting the findings. First, as a single-center observational study, our results reflect local patterns of maternal substance use, prenatal care access, and treatment availability, which may not be generalizable to other regions with differing socioeconomic and healthcare contexts. Second, maternal substance use was identified through self-reporting and clinical indications for toxicology testing, which may have led to the underestimation of exposure due to reporting bias or inconsistent testing practices. Third, our study included only NICU-admitted infants, excluding term infants with cocaine exposure who remained in the well-baby nursery. This limitation prevented the inclusion of a cocaine-only comparison group, which could have provided additional insight into the independent effects of cocaine exposure. Fourth, while we observed lower birth and discharge weights among infants in the opioid-with-cocaine (OwC) group, we did not assess small-for-gestational-age (SGA) status or adjust for gestational age in our birth weight analysis. This omission may confound interpretation, as lower birth weights in the OwC group could be attributable to higher rates of preterm birth rather than an independent association. Future studies should categorize birth weight by gestational age to more accurately assess fetal growth restrictions. Fifth, we were unable to account for other maternal health conditions—such as obesity and diabetes—that are known to influence birth outcomes and early postnatal weight change. The absence of these data represents an important limitation, as highlighted in the prior literature [42]. Lastly, as noted in our discussion, the neurobehavioral effects of cocaine exposure may mimic symptoms of NOWS, and treatment is typically guided by symptom severity rather than etiology.

## Figures and Tables

**Table 1:** Maternal demographics.

	Opioids with Cocaine (OwC) *N* = 108	Opioids without Cocaine (OwoC) *N* = 234	*p* Value
Maternal Age, Years	30 ± 5	29 ± 5	0.41
Race			0.40
Black	71 (66)	134 (57)	
White	35 (33)	95 (41)	
Hispanic	1 (1)	4 (2)	
Education Level			0.22
Less than High School	29 (27)	52 (22)	
High School Degree	53 (49)	128 (55)	
Some College or Post-Graduate	17 (16)	45 (19)	
Marital Status			0.18
Single, Never Married	101 (94)	198 (85)	
Married	5 (5)	26 (11)	
Insurance			0.12
Medicaid	90 (83)	210 (90)	
None	16 (15)	18 (8)	
Prenatal Care	69 (64)	192 (82)	<0.01
Cesarean Delivery	40 (36)	105 (44)	0.20
Gravida	4 ± 2	4 ± 2	0.33
Para	3 ± 2	2 ± 2	0.05
Parity 1	19 (20)	54 (27)	0.30
Parity 2–3	42 (44)	90 (45)	
Parity ≥ 4	34 (36)	56 (28)	
Maternal Hepatitis C			0.40
Not Tested	54 (49)	113 (47)	
Positive	25 (23)	45 (19)	
Maternal Psychiatric History	32 (29)	69 (28)	0.90
Maternal Chronic Pain History	3 (5)	31 (29)	<0.01
Substance Use			
Amphetamine	25 (23)	41 (17)	0.24
Heroin	39 (36)	61 (25)	0.06
Fentanyl, Illicit	41 (37)	36 (15)	<0.01
Tobacco	66 (60)	117 (48)	0.05
Marijuana	73 (66)	119 (50)	<0.01
Benzodiazepine	36 (33)	64 (26)	0.25
Polysubstance, Any	108 (100)	198 (82)	<0.01
Number of Polysubstance	5 ± 1	3 ± 1	<0.01
MOUD, Any	29 (26)	94 (39)	0.03

MOUD: medicine for opioid use disorder. Data are presented as mean ± SD and/or *N* (%) number of infants and their percentage based on total number of infants with complete data.

**Table 2:** Infant demographics and outcomes.

	Opioids with Cocaine (OWC) *N* = 110	Opioids without Cocaine (OWoC) *N* = 243	*p* Value
Gestational age, weeks	36 ± 4	36 ± 3	0.42
Birth weight, grams	2512 ± 712	2656 ± 763	0.09
NBW (≥2500 g)	63 (57)	157 (65)	
LBW (1500–2499 g)	35 (32)	67 (28)	
VLBW (1000–1499 g)	8 (7)	9 (4)	
ELBW (<1000 g)	4 (4)	10 (4)	
Male sex	49 (45)	121 (50)	0.42
Birth head circumference, cm	31 ± 3	32 ± 3	0.16
Length of stay, days	28 ± 22	21 ± 21	<0.01
Preterm birth (<37 weeks)	52 (47)	89 (37)	0.06
Discharge weight, grams	2619 ± 773	3060 ± 810	<0.01
Feeding problems, any	66 (27)	40 (37)	0.08
NOWS ∞	62 (56)	85 (35)	<0.01
Management of NOWS			
Morphine only	36 (33)	61 (25)	0.16
Morphine treatment, days	27 ± 14	22 ± 15	0.88
Phenobarbital	26 (24)	24 (10)	<0.01
Phenobarbital start DOL	5 ± 6	8 ± 9	0.17
Head ultrasound abnormal *	23 (31)	35 (32)	1.00
Discharge home with mother	14 (13)	158 (65)	<0.01

DOL: days of life; ELBW: extremely low birth weight; NOWS: neonatal opioid withdrawal syndrome, requiring treatment; NBW: normal birth weight; LBW: low birth weight; VLBW: very low birth weight. ∞ represents initiation of NOWS treatment. * Any grade intraventricular hemorrhage or periventricular leukomalacia. Data are presented as mean ± SD and/or *N* (%) number of infants and their percentage based on total number of infants with complete data.

## Data Availability

The data presented in this study are available on request from the corresponding author due to (privacy, legal or ethical reasons due to sensitive nature of data).

## Supplementary Materials

The following supporting information can be downloaded at: https://www.mdpi.com/article/10.3390/children12070916/s1, Table S1: Length of Hospital Stay by prematurity and NOWS Treatment.
