# Supplementary material for: Mothers with Concurrent Opioid and Cocaine Use and Neonatal Opioid Withdrawal Syndrome
Source: Children (Basel). 2025 Jul 11;12(7):916. doi: 10.3390/children12070916 (PMC12293442; doi:10.3390/children12070916)
Supplement: Supplementary file 1 [file children-12-00916-s001.zip › children-3710154-supplementary.pdf]

Supplemental Table S1: Length of Hospital Stay by prematurity and NOWS Treatment

	**Term Infants (≥ 37 weeks)**			**Preterm Infants (< 37 weeks)**		
	Opioids with Cocaine	Opioids without Cocaine	P value	Opioids with Cocaine	Opioids without Cocaine	P Value
**LOS, Days**	25 ± 21	18 ± 17	0.02	32 ± 22	26 ± 26	0.19
**LOS with NOWS**	34 ± 21	31 ± 17	0.54	32 ± 15	22 ± 11	0.02
